# NASICON Membrane with High Ionic Conductivity Synthesized by High-Temperature Solid-State Reaction

**DOI:** 10.3390/ma17040823

**Published:** 2024-02-08

**Authors:** Mihaela Iordache, Anisoara Oubraham, Irina Petreanu, Claudia Sisu, Simona Borta, Catalin Capris, Amalia Soare, Adriana Marinoiu

**Affiliations:** National R&D Institute for Cryogenics and Isotopic Technologies—ICSI Ramnicu Valcea, Uzinei No. 4, 240050 Vâlcea, Romania; mihaela.iordache@icsi.ro (M.I.); irina.petreanu@icsi.ro (I.P.); claudia.sisu@icsi.ro (C.S.); simona.borta@icsi.ro (S.B.); catalin.capris@icsi.ro (C.C.); amalia.soare@icsi.ro (A.S.); adriana.marinoiu@icsi.ro (A.M.)

**Keywords:** NASICON ceramic membranes, solid state reaction, ionic conductivities

## Abstract

In the present work, we studied the impact of excess Na addition on the structure of the standard NASICON ion conductor along with Na ion transport mechanisms. In this sense, NASICON ceramic membranes (NZSP) were prepared by a simple chemical synthesis method, the solid state reaction (SSR), using an excess of 5% Na (Na_3.15_Zr_2_Si_2_PO_12_) and an excess of 10% Na (Na_3.3_Zr_2_Si_2_PO_12_), in order to improve the conduction properties of the ceramic membrane. The characterization of the NZSP nanoparticles was performed by measuring the particle size by dynamic light scattering (DLS), the morphology of the NASICON samples pre-sintered at 1100 °C was analyzed by the SEM method (scanning electron microscope), and X-ray diffraction (XRD) analysis was used to investigate the crystal structure of samples, while the surface area was measured using the BET technique. The electrical properties (i.e., ionic conductivity) were evaluated by impedance spectroscopic methods at room temperature (RT). Following the experiments for NASICON membranes without Na excess, with 5% Na excess, and with 10% Na excess synthesized at different pressing forces and sintering temperatures, it was found that membranes with a 10% Na excess, sintered at 1175 °C for 10 h, presented a good ionic conductivity (4.72 × 10^−4^ S/cm).

## 1. Introduction

The necessity to reduce the greenhouse gases resulting from fossil fuel combustion along with the depletion of fossil fuel reserves requests a drastic change to a non-carbonaceous economy. In recent years, socio-economic progress has involved the excessive and irrational use of energy resources, especially non-renewable ones. The need for a clean environment, which can only be achieved through sustainable development, involves alternatives for both energy sources and the ways of their production and utilization. This new paradigm involves increasing the weight and diversity of renewables. The development and use of these energy sources can better satisfy the function of energy conversion and storage [1,2]. Many solutions regarding energy production and storage technologies, including a variety of fuel cells as eco-friendly energy sources, or battery and capacitors for energy storage systems, are still developing. Studies have shown that sodium ion batteries (SIBs) could be more competitive than lithium ion batteries (LIBs) due to large-scale energy storage applications and due to the large reserve of sodium in nature. Therefore, solid sodium ion electrolytes are increasingly analyzed due to their potential use in solid-state sodium metal batteries, which show a high safety and energy density [3,4,5,6].

In electrochemical applications, two types of electrolytes are used: liquid and solid. The first ones are more convenient to use in various applications. In addition, they are more competitive in terms of cost. Instead, the solid ones have other advantages, such as: non-flammable, non-volatile, non-toxic, non-corrosive, and thermally stable over a wide range of temperatures. Characteristics such as a low ionic conductivity and weak electrolyte–electrode interaction are obstacles in the large-scale development of all-solid-state batteries (ASSBs). In accordance with the specialized literature, the development of these types of batteries still requires investigation regarding the transport phenomena of solid electrolytes and the elements regarding their structure [7]. In addition to the actual use in batteries, solid electrolytes can also be used in other types of equipment: sensors, fuel cells, supercapacitors, etc., [8]. The solid electrolytes used in the ASSBs batteries must have similar technical conditions to those of liquid electrolytes: ionic and electrical conductivity, stability, number of charge–discharge cycles, etc., [9]. In the context where energy storage tends to become the critical component of the energy system, rechargeable batteries are taken into account to increase its reliability but also for the most efficient conversion of various energy sources. Although currently Li-ion batteries are used on a very large scale in various electrochemical devices, for some time the scientific community has begun to pay more and more attention to Na-ion type batteries, due to their low cost, abundance, and non-toxicity. One of the key components of Na-ion batteries are solid-state electrolytes (SSEs). By comparison, conventional liquid electrolytes still have deficiencies in terms of flammability and leakage. On the other hand, SSEs have improvement potential in terms of durability and safety, as well as the constructive simplicity of the design of future Na-ion batteries. Finally, the scientific literature adds the fact that this type of battery (solid-state NIBs) with a high-voltage metallic sodium anode and cathode have the potential for much higher energy densities than conventional batteries based on liquid electrolytes [10,11,12].

The safest and most cost-competitive batteries have proven to be solid-state sodium ion batteries with a Na_3_Zr_2_Si_2_PO_12_ (NZSP) ceramic electrolyte. Studies have demonstrated that this type of electrolyte shows promising ionic conductivity for a high-temperature solid electrolyte, even if, under normal conditions (ambient temperature), the sodium ion conductance is lower than that of organic electrolytes. Therefore, improving the ionic conductivity of NZSP will lead to an increase in the power density of sodium batteries for both high temperatures and under normal conditions [13].

Several research studies have already been carried out on the Na_1+x_Zr_2_Si_x_P_3−x_O_12_ (NZSP) family to produce sodium-ion-based solid electrolytes by optimizing the Na dosage using different preparation methods such as solid-state reaction (SSR), solution-assisted solid-state reaction (SASSR), SSR using nanoparticle precursors, spark plasma sintering, and sol-gel synthesis. These studies reported an interdependence between the total ionic conductivity and the synthesis conditions. The conditions refer especially to the final sintering temperature, with direct implications on the density, purity, microstructure, crystallinity, and homogeneity of the composition. The most eloquent example is NZSP with x = 2 (Na_3_Zr_2_Si_2_PO_12_), which indicates a good overall ionic conductivity [14].

NASICON ceramic membranes are isomorphous compounds with a 3D structure, with high ionic conductivity at high temperatures, comparable to that of a liquid electrolyte. They can be prepared as monocrystalline/polycrystalline ceramics, thin films, or glass. Ceramic membranes of the NASICON type are used especially as a solid electrolyte in a sodium ion battery (it has high ionic conductivity), in precision instruments (it has a low coefficient of thermal expansion < 10^−6^ K^−1^), in the detection of CO_2_, SO_2_, NO, NO_2_, NH_3_ and H_2_S gases (their electrical conductivity is sensitive to these molecules), in various catalysis processes, in the immobilization of radioactive waste, and in the removal of sodium from water [15,16,17].

Na_1−x_Zr_2_Si_x_P_3−x_O_12_ (NZSP) structured with NASICON was designed for the first time and reported by Hong and Goodenough et al. and presents an ionic conductivity of about 10^−4^ S cm^−1^ at ambient temperature. Since then, this NASICON structure has attracted much attention due to its superior stability against water and air and wide electrochemical window against Na metal [18,19].

The sodium superionic conductor commercially known as NASICON, with a general chemical formula Na_1+x_Zr_2_Si_x_P_3−x_O_12_, 0 ≤ x ≤ 3, seem to be one of the promising solid electrolytes, with a tridimensional diffusion network. This physical property allows both the sodium ion species active transport (i.e., the ionic conductor) and the blocking of electrons between the anode and cathode compartments of the cells [20]. NASICON is known for both a high ionic conductivity and relatively high chemical stability. The structure of this material (NASICON) is arranged in the form of a rigid three-dimensional network with octahedral structures of zirconium oxides (ZrO_6_) and tetrahedral structures of phosphate and silicon oxides (PO_4_^3−^/SiO_4_^4−^). The crystalline network of this material, through the interconnecting channels, ensures an adequate conductivity for sodium ions. Although Na_3_Zr_2_Si_2_PO_12_ exhibits a good ionic conductivity (10^−4^ S/cm) at room temperature, in recent years, efforts have been made to increase the ionic conductivity for a good electrochemical performance. Among Na-ion-conducting SSEs, the sodium superionic conductor (NASICON) with a general formula of Na_1+x_Zr_2_Si_x_P_3−x_O_12_ (1.0 ≤ x ≤ 3) has attracted the most attention due to its high ionic conductivity and low thermal expansion [21,22]. The NASICON type solid electrolyte is characterized by chemical stability suitable for electrochemical applications in different working environments that involve either moist air or the presence of aqueous solutions. Characteristics that add to the ionic conductivity have been discussed previously. The accumulation of these properties with excellent values make this product one of the most promising solid electrolytes that use Na^+^ ions. On the other hand, the presence of ZrO_2_ as an impurity predisposes the volatilization of Na and P elements when the material is obtained by sintering at a high temperature. This effect can be combated by the excessive and controlled dosage of Na and P in the manufacturing raw materials, and the appearance of ZrO_2_ impurities along the crystal lattice can be avoided or minimized; in this way, the mobility of sodium ions can no longer be disturbed. In recent years, studies have focused on improving the ion conduction properties of NASICON, exploring the mechanism of ion conduction and the underlying strategies to form a stable interface with electrode materials [22,23].

The novelty of this work lies in studying the impact of excess Na addition on the structure of the standard NASICON ion conductor together with Na ion transport mechanisms. In view of this, a simple but effective method, chemical synthesis of the solid-state reaction (SSR), was used to prepare solid electrolytes of the Na_1+x_Zr_2_Si_x_P_3−x_O_12_ type, with a high ionic conductivity, using sodium in excess. The precursors used in the synthesis of Na_1+x_Zr_2_Si_x_P_3−x_O_12_ had particles at the nano level, which led to a major advantage in the microstructure, crystalline structure, and electrical performance. To investigate the effect of excess sodium on the properties of the material, as well as its loss during the sintering process due to possible evaporation, a material was prepared with an excess of 5% and 10% sodium by weight, with respect to the stoichiometric composition. Recently, solid-state electrolytes have been considered as an alternative to liquid electrolytes, because they are safer and have a good durability [19].

The purpose of this work is to prepare NASICON ceramic membranes with a high ionic conductivity, by the solid-state reaction chemical synthesis method, using an excess of 5% Na (Na_3.15_Zr_2_Si_2_PO_12_) and an excess of 10% Na (Na_3.3_Zr_2_Si_2_PO_12_).

## 2. Materials and Methods

### 2.1. Precursor Materials and Synthesis Method

**Figure 1:**
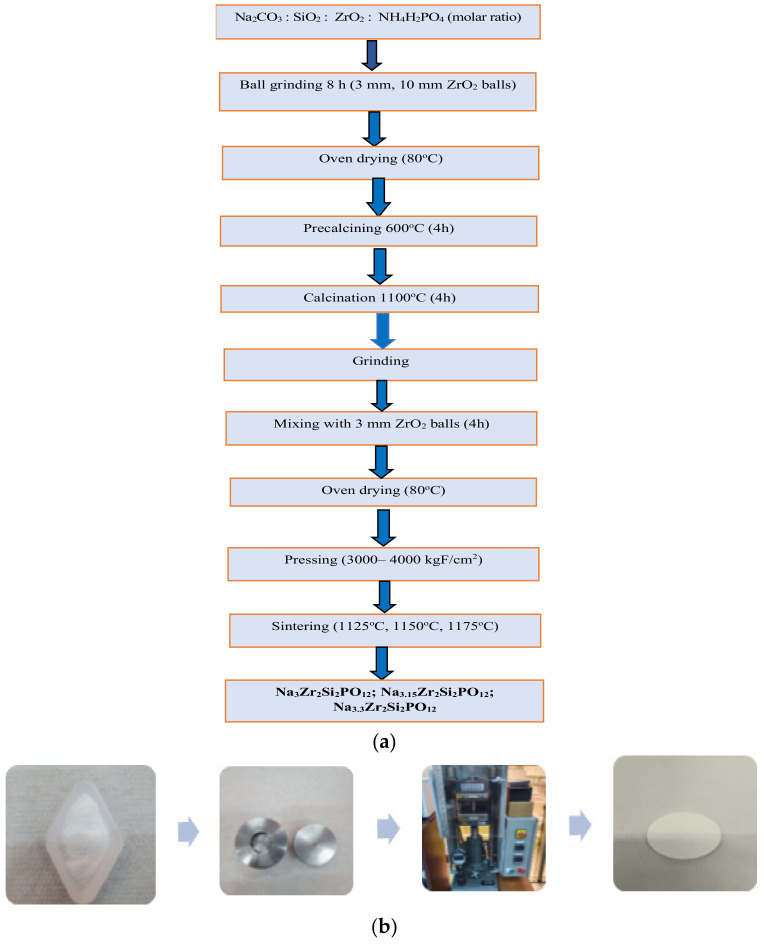
(**a**) Method of obtaining the NASICON membrane; (**b**) NASICON ceramic membrane preparation process.

To obtain the ceramic membranes, a solid-state reaction process was used, through the stoichiometric combination of Na_2_CO_3_ (98%, Alfa Aesar, Haverhill, MA, USA), SiO_2_ (Umicore), ZrO_2_ (99%, Sigma-Aldrich, Saint Louis, MO, USA), and NH_4_H_2_PO_4_ (Scharlab, Barcelona, Spain), in a molar ratio of 1.5:2:2:1 for the membrane without excess Na (Na_3.0_Zr_2_Si_2_PO_12_) (NZP), in a molar ratio of 1.575:2:2:1 for the membrane with an excess of 5% Na (Na_3.15_Zr_2_Si_2_PO_12_) (NZP5), and in a molar ratio of 1.65:2:2:1 for the membrane with an excess of 10% Na (Na_3.3_Zr_2_Si_2_PO_12_) (NZP10). The precursors were mixed with ethanol and subjected to grinding in the planetary ball mill. Thus, the precursors were mixed together with ethanol in a ZrO_2_ container and ground in a ball mill for 12 h, with ZrO_2_ balls of 10 mm and 3 mm in diameter, at 300 rpm. The homogeneous material obtained after grinding was dried in an oven at 80 °C, precalcined at 600 °C for 4 h, then calcined at 1100 °C for 4 h. Finally, the material calcined at 1100 °C was mixed in the presence of ethanol, in a ball mill, at 300 rpm, for 4 h, using zirconium balls with a diameter of 3 mm. After this stage, the obtained material was subjected to oven drying at a temperature of 80 °C. The obtained powder was pressed by cold at room temperature, isostatic pressing into pellets, for 5 min, using a hydraulic press. The size of the pellets is approx. 20 mm and the thickness is approx. 1.0 mm. The sintering of the manufactured pellets was carried out at three temperatures (1125 °C, 1150 °C, 1175 °C), with a heating rate of 5 °C min^−1^, for 10 h, in a tubular furnace, in a nitrogen gas environment, to remove any volatile species (Figure 1).

All laboratory-synthesized samples were compared to a standardized commercial NASICON membrane (NZSP, 4TOONE Ltd., Ulsan, Republic of Korea).

### 2.2. Equipment and Characterization Method

The characterization of NZSP nanoparticles was performed by measuring particle size by dynamic light scattering (DLS), using the Nano DS Dual Light Scattering Particle Size Analyzer (Cilas, Orléans, France). Dynamic light scattering uses Brownian motion to measure the size of nanoparticles. The crystal structure of the NZSP samples was characterized by XRD, performed on a Rigaku MiniFlex 600 X-ray diffractometer equipped with a CuK-a X-ray source (1.541838 Å); samples were scanned from 10° to 60° at a high resolution (scan step 0.01°, scan speed 1.0° min^−1^), using a monochromator to suppress noise levels. The morphology of the NASICON samples pre-sintered at 1100 °C was analyzed using the field emission scanning electron microscope variable pressure (FESEM VP) brand Carl Zeiss (Jena, Germany).

To measure the surface, the Autosorb IQ analyzer (Quantachrome, Boynton Beach, FL, USA) was used, using the Brunauer–Emmett–Tellerbet technique. The nitrogen temperature at which the adsorption -desorption isotherms were obtained was 77 K. Before being analyzed, the samples were degassed at a temperature of 115 °C for 4–6 h. To calculate the specific surface, the Brunauer–Emmett–Teller (BET) equation was used, using the criterion of linearity in the range 0.1–0.3 P/P0. The investigation of the pore distribution was carried out using the equations corresponding to the theory of the Barrett–Joyner–Halenda (BJH) method using the desorption isotherm.

A broadband dielectric spectroscopy (BDS) measurement was performed with an Alpha-A modular measurement system from NOVOCONTROL (Montbauer, Germany), equipped with a Quattro Cryosystem temperature controller with the temperature stability more than 0.1 K. The samples were scanned isothermally over a frequency range from 10^−1^ to 10^7^ Hz using an AC signal with an amplitude of 0.1 V, for different temperature points, in 10 K steps, from −60 to 100 °C. Before scanning, each sample was annealed at 120 °C for two hours, under nitrogen flow, inside the measurement cell to remove the residual moisture and volatiles.

## 3. Results and Discussion

### 3.1. Physico-Chemical Characterization of the Material

**Figure 2:**
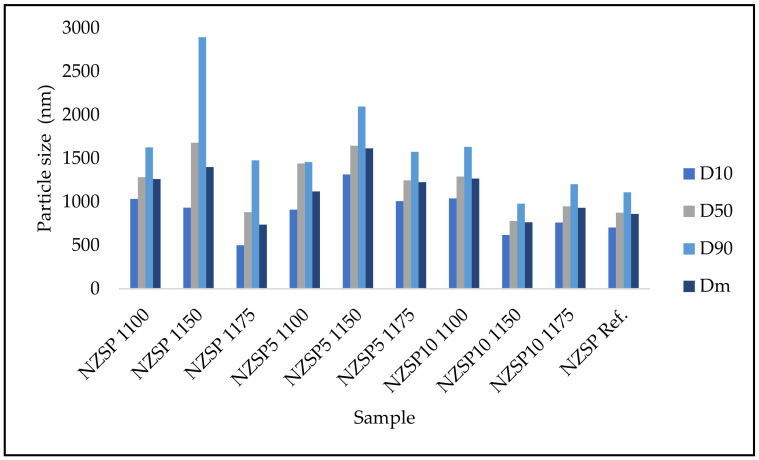
Particle size distribution for NASICON membranes.

The analysis of particle size and its distribution is important for determining the properties of particulate materials. It was found that the size of the particles is important in the conductivity of crystalline electrolytes. It was also observed that the reduction in the particle size favors a good ionic conductivity. In the case of our study, the particles were reduced in size and gradually homogenized, due to the application of a strict and very energetic ball mill grinding. After finishing the ball milling, the obtained mixture has an average particle size of microns (~2000 nm). After the sintering process, the particles have larger diameters (<3000 nm) due to thermally induced aggregation (Figure 2). Table 1 shows that 10% of the NASICON powder particles sintered at 1100 °C have a size of approx. 1000 nm, while for NASICON sintered at 1150 °C the particles have a size between 618.6 and 1315.4 nm, and for NASICON sintered at 1175 °C the particles have a size between 500.6 and 1008.5 nm. Dm corresponds to the modal diameter of the distributions; D_50_ corresponds to the median. From the data obtained, it is observed that during sintering a change in the texture of the NASICON composition occurs as a direct consequence of the high sintering temperature. As the sintering temperature increases, the particle size decreases due to additional grinding steps during sintering, which helped to reduce the particle size of the precursors which mitigated the risk of aggregate formation during heat processing [24]. 

**Figure 3:**
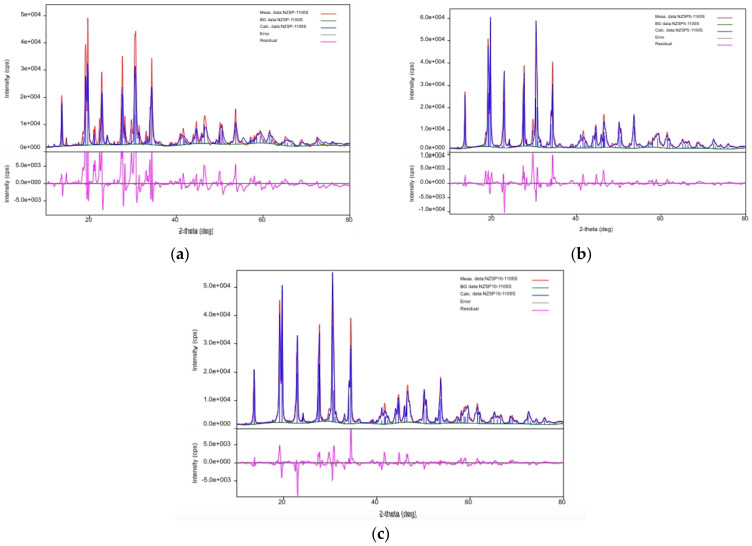
X-ray diffraction (XRD) of (**a**) NZSP, (**b**) NZSP5, and (**c**) NZSP10 sintered at 1100 °C.

X-ray diffraction (XRD) analysis (Figure 3) was used to investigate the crystal structure of samples of Na_3_Zr_2_Si_2_PO_12_, Na_3.15_Zr_2_Si_2_PO_12_, and Na_3.3_Zr_2_Si_2_PO_12_ materials, sintered at 1100 °C. From the XRD investigations for the samples NZSP, NZSP5, and NZSP10, the following phases were identified: for NZSP, a phase corresponding to Na_3_O_12_PSi_2_Zr1_.93_ (98.4%) and a secondary phase ZrO_2_ (1.71%); for NZSP5, a phase corresponding to Na_3.17_ O_12_P_1.09_Si_1.91_Zr_1.93_ (99.66%) and as secondary phase Zr O_2_ (0.34%); while for NZSP10, the phase corresponding to Na_3.17_Zr_1.93_Si_1.91_P_1.09_O_12_ (98.2%) and as secondary phase ZrO_2_ (1.8%). The NZSP respective ZrO_2_ content were calculated based on the reference intensity ratio method (RIR). To determine the lattice parameters for the samples NZSP, NZSP5, and NZSP10, the angular correction of the lattice constants after Rietveld refinement was used as follows: a = 15.63; 15.639; 15.64 Å, b = 9.044; 9.047; 9.047 Å, c = 9.214 Å; 9.226; 9.227, V = 1081.3; 1085.2; 1085.9 Å^3^, α = y = 90.0°, β = 123.95°; 123.77°; 125.74° for phases NZSP, NZSP5 and NZSP10; and a = 5.148; 5.254; 5.218 Å, b = 5.207; 5.389; 5.104 Å, c = 5.315; 5.86; 5.375 Å, V = 140.73; 146.68; 137.9 Å^3^, α = γ = 90.0°, β = 99.09°; 101.66°; 105.55° for zirconium. The following raw mesh parameters were used for comparison: a = 15.64 Å, b = 9.05 Å, c = 9.210 Å, V = 1085.1 Å^3^, α = γ = 90.0°, β = 123.72° for NZSP; and a = 5.148 Å, b = 5.202 Å, c = 5.323 Å, V = 140.7 Å^3^, α = γ = 90.0°, β = 99.16° for zirconium [25]. The calculated densities recorded results comparable to those in the literature, 3.27 g/cm^3^ for Na_3_Si_2_Zr_2_PO_12_ and 5.889 g/cm^3^ for zirconium. NZSP recorded a density value of 3.218 g/cm^3^, while for zirconium it was 5.815 g/cm^3^; NZSP5 recorded a density value of 3.229 g/cm^3^, while for zirconium it was 5.812 g/cm^3^; and NZSP10 recorded a density value of 3.227 g/cm^3^, while for zirconium it was 5.771 g/cm^3^.

The microstructural and elemental investigations were carried out using a field emission scanning electron microscope with auto electronic emission at variable pressure (field emission scanning electron microscope variable pressure—FESEM VP) from the brand Carl Zeiss. The microscope is equipped with an EDS (energy dispersive X-ray spectroscopy) system that allows the quantitative and qualitative analysis of all chemical elements up to B (Z = 5).

**Figure 4:**
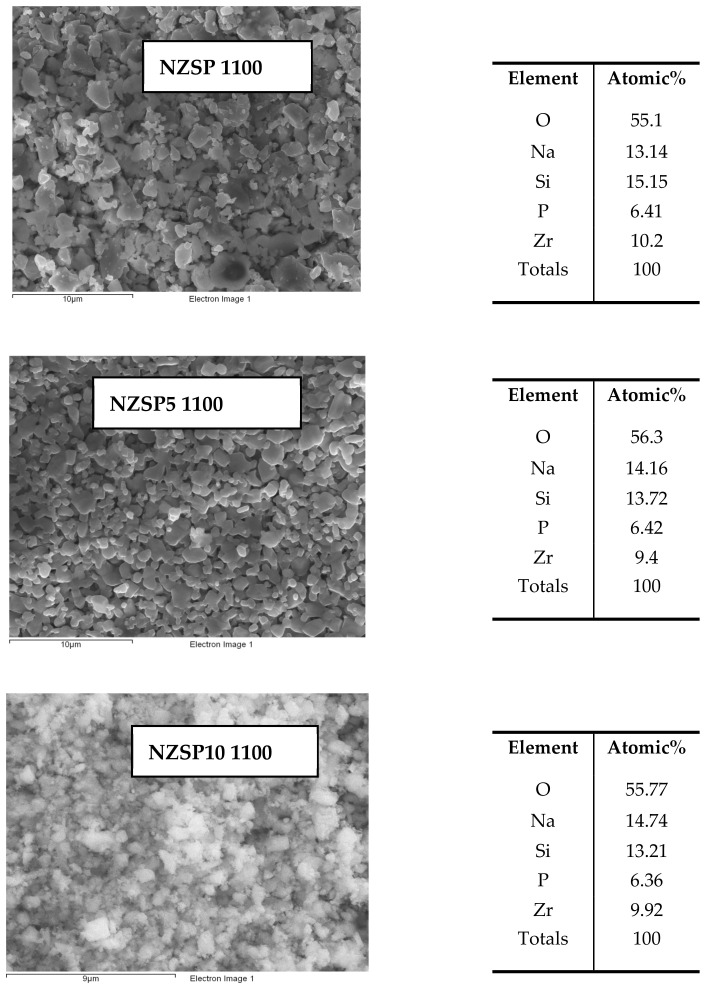
EDX analysis for NZSP, NZSP5, and NZSP10 pre-sintered at 1100 °C (At %).

SEM images of the pre-sintered NASICON surfaces are shown in Figure 4. The micrographs show the arrangement and close packing of the molten particles, as well as the NZSP particles with smoothed grains and diameters in the micron to submicron range, as well as the appearance of mesopores in the network, through the unoccupied spaces between particle assemblies. To explore the chemical composition of the NZSP samples, an energy spectrum test was performed on the samples of Na_3_Zr_2_Si_2_PO_12_, Na_3.15_Zr_2_Si_2_PO_12_, and Na_3.3_Zr_2_Si_2_PO_12_, pre-sintered at 1100 °C. The chemical composition is composed of the elements Na, Zr, Si, P, and O and the calculated atomic ratios of Na, Si, and O were: 13.14:15.15:55.1; 14.16:13.72:56.3; 14.74:13.21:55.77, respectively, which are close to the theoretical atomic ratio (15:10:60). EDX analysis indicated a homogeneous distribution of elements which is observed from EDX mappings. From the EDS analysis, it can be seen that the content of Zr and P remains approximately constant for all three samples. However, a higher content of Zr and a lower content of P are found. These changes may be due to the location of the two elements on layers (La, 2.04 KeV) for Zr and (Ka, 2.02 KeV) for P, which are adjacent in the EDS energy spectrum [1]. It turned out that the sum of the molar ratios of Zr and P elements is approximately equal to the sum of the theoretical molar ratios (15%). Studies have shown that during conventional sintering (at about 1000 °C), there is a saturated vapor pressure of Na and P elements above the sample that prevents further vaporization and also maintains the stability of the NZSP solid electrolyte [25].

**Figure 5:**
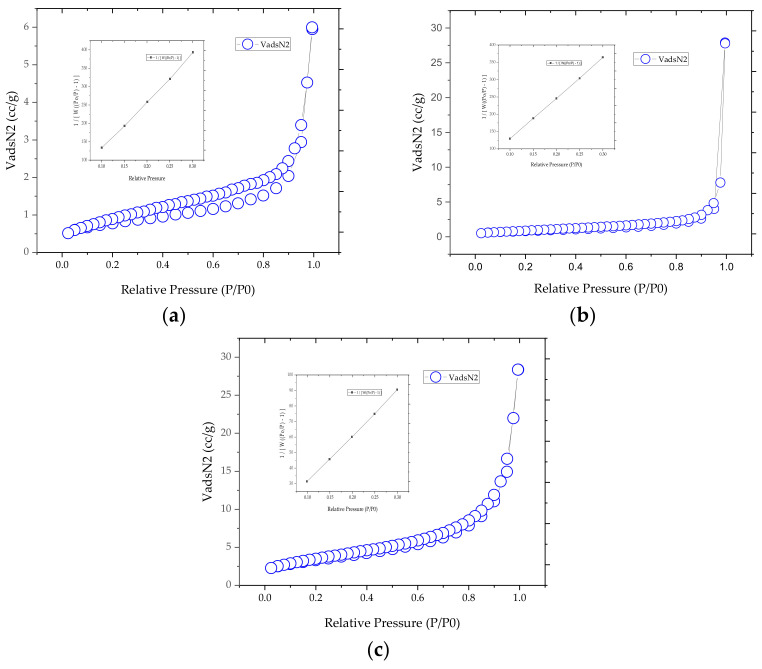
BET adsorption and desorption isotherms, with reference to NASICON powder: (**a**) NZSP, (**b**) NZSP5, and (**c**) NZSP10.

Figure 5 shows the results of the specific surface area and the microporosity of the NZSP samples, which were performed by BET and BJH analysis, respectively. BET analysis is based on physical adsorption between nitrogen gas molecules and the exposed surface of the NZSP material, at liquid nitrogen temperature. BJH textural analysis is shown in Table 2. Surface analysis was performed using BET analysis (Figure 4). Nitrogen adsorption is caused by the existence of intrinsic surface energies. NZSP10 powder showed a BET surface area of 11.78 m^2^/g compared to a surface area of 2.94 and 2.69 m^2^/g for NZSP5 and NZSP, respectively. The curves obtained from the nitrogen adsorption/desorption isotherms indicated a hysteresis loop, suggesting the presence of a predominantly mesoporous structure.

### 3.2. Testing Electrical Properties of NASICON Ceramic Membrane

Dielectric characterization provides important information about molecular relaxation at different temperatures and the influence of the variable electrical field (variable frequency) on it. In this respect, specific parameters such as relative permittivity (also denoted as the dielectric constant), with the two components of the real (*ε*′) and imaginary (*ε*″) parts, are recorded, when the sample is subject to the frequency sweep on a large range (10^−1^–10^7^ Hz), at different temperatures. When an alternating current is applied through the sample, the charge carriers become polarized with a delay. This delay causes a loss of energy in the form of heat which is called dielectric loss (expressed as tan *δ*). Dielectric loss in the material combines with dielectric relaxation and electrical conduction effects. Electrical permittivity varies with the temperature, frequency, orientation, pressure, and molecular structure of materials. The total dielectric constant (*ε_r_*) was expressed as a function of the capacitance of a capacitor (Equation (1)) or as a complex parameter composed of a real part (*ε*′*_r_*) and an imaginary part (*ε*″*_r_*) (Equations (2a) and (2b)).

(1) $$\varepsilon_{r}\left(\omega \right)=\frac{C_{p}}{C_{0}}=\frac{C_{p}d}{\varepsilon_{0}A}$$

(2a) $${\varepsilon \prime }_{r}\left(\omega \right)=\left|\varepsilon_{r}\left(\omega \right)\right|cos\delta =\left|\varepsilon_{r}\left(\omega \right)\right|sin\theta$$

(2b) $${\varepsilon \prime \prime }_{r}\left(\omega \right)=\left|\varepsilon_{r}\left(\omega \right)\right|sin\delta =\left|\varepsilon_{r}\left(\omega \right)\right|cos\theta$$

where *C_p_* represents the capacitance measured on the parallel faces, *C*_0_ represents the capacitance of the vacuum, *C*_0_ = *ε*_0_*A*/*d*, where *ε*_0_ represents the permeability of the vacuum, *A* is the transverse surface area of the capacitor between the plates of which the dielectric material is located, and *d* represents the thickness of the dielectric layer; *ω* = 2*πf* is the angular frequency of the applied field, and *δ* = 90 − *θ*, where *θ* is the phase angle.

The samples of NASICON synthesized in the laboratory (NZSP, NZSP5, NZSP10), sintered at different temperatures of 1100, 1150, and 1175 °C were analyzed by the broadband dielectric spectroscopy (BDS) method.

**Figure 6:**
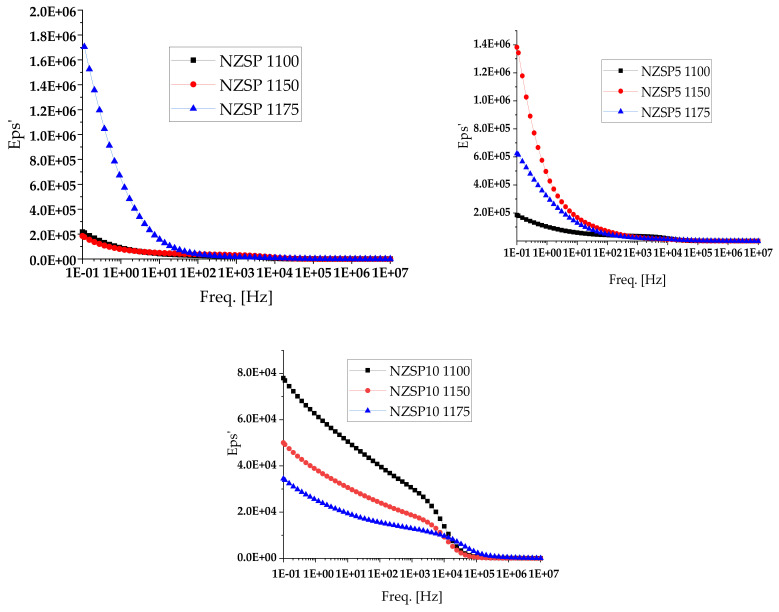
Dielectric constant variation in NZSP, NZSP5, and NZSP10 samples at different frequencies, at room temperature (RT).

Figure 6 shows the dependence of the dielectric constant as a function of the frequency *ε*^+^ (*ω*), at room temperature for the analyzed samples. It is observed that the dielectric constant values decrease with increasing frequency. Dielectric relaxation can occur in materials due to imperfections such as vacancies or concentrated electronic charges. Also, the increase in the dielectric constant at low frequencies can be explained by space charge polarization mechanisms. According to this model, the charge carriers follow the applied field at low frequencies, leading to increased polarization and implicitly to high permittivity values. Conversely, at high frequencies the charge carriers cannot follow the applied field because the period is much too short and the field changes direction before the charges align with the direction of the field; hence, the net bias decreases and so does the electric constant.

**Figure 7:**
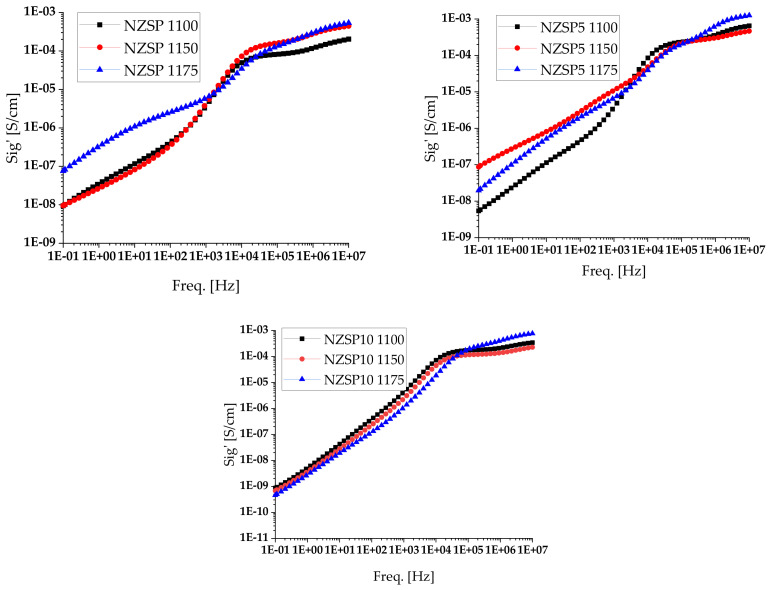
Variation in conductivity of NZSP, NZSP5, and NZSP10 samples at room temperature (RT).

From the analysis of broadband dielectric spectroscopy data, the direct current ionic conductivities (σ) of the analyzed samples were calculated at room temperature (Table 3) and the variation of conductivity was graphically represented as a function of temperature (Figure 7). The measured conductivity values are comparable in order of magnitude to those obtained for commercial NASICON. After analyzing the experimental data, it was observed that the samples of NASICON sintered at 1150 °C and at 1175 °C had higher ionic conductivity values than the commercial one. We can also note that the higher the sintering temperature, the higher the ionic conductivity of the membranes.

**Figure 8:**
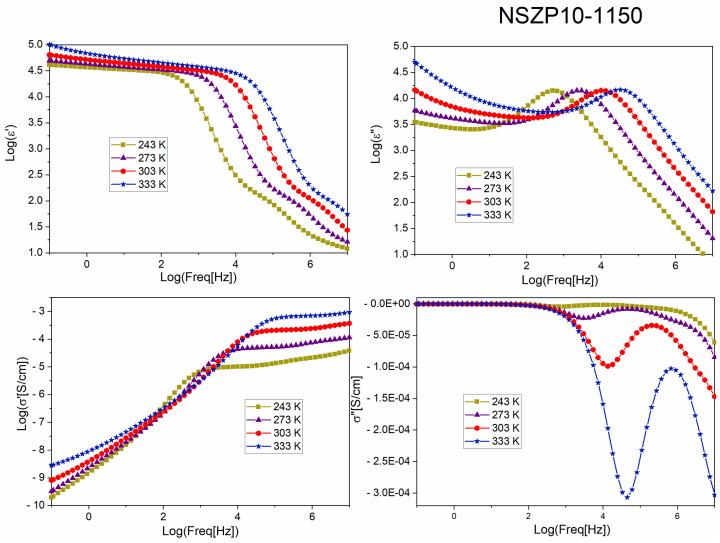
Variation in the real permittivity (ε′) and imaginary permittivity part (ε″), depending on frequency (logarithmic mode) and the graphs of real (σ′) and imaginary (σ″) conductivity, function of frequency, recorded for the NZSP10-1150 sample, at different temperatures (−30, 0, 30, and 60 °C).

**Figure 9:**
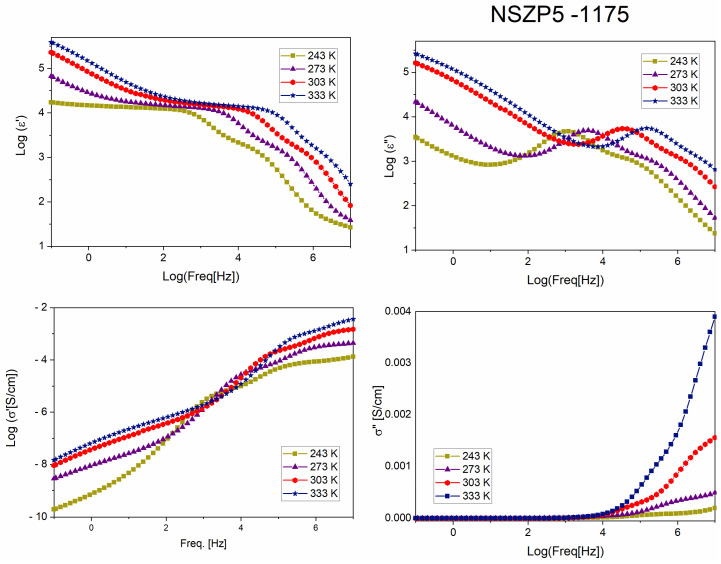
Variation in the real permittivity (ε′) and imaginary permittivity part (ε″), depending on frequency (logarithmic mode) and the graphs of real (σ′) and imaginary (σ″) conductivity, function of frequency, recorded for the NZSP5-1175 sample, at different temperatures (−30, 0, 30, and 60 °C).

In Figure 8, some parameters determined by BDS are presented, such as the dielectric permittivity, the real (ε′) and the imaginary (ε″) parts, depending on the frequency. Functions are presented in logarithmic mode. The real and imaginary parts of the conductivity (σ′ and σ″) variation function of the logarithm of frequency in logarithmic representation are show in Figure 8, too. We can notice that the real part of permittivity (ε′) decreases steeply at a higher frequency and the imaginary part (ε″) shows a maximum at half of the frequency range. Moreover, the value of ε′ increases with temperature and the maximum of ε″ (freq.) function shifts to a higher frequency temperature. From the graph of real conductivity (σ′), we can observe an increase in the conductivity σ′ (freq) values from lower to medium frequencies, up to a constant value. The value of the real part of the conductivity (σ′) at the beginning of the plateau represents the direct current (DC) conductivity or the ionic conductivity of the material [26]. It can be noticed that the ionic conductivity of the sample increases with the temperature. The curves of the imaginary part of the conductivity depending on frequency shows a minimum, which goes deeper with an increasing temperature. Figure 9 presents the variation in the same dielectric parameters, with frequency, for the sample with a 5% Na excess, sintered at 1175 °C. The curves of the real and imaginary parts of permittivity present almost the same trend as the NZSP10-1150 sample, and the differences for the real part of the conductivities are not major. The graphs of the imaginary part of the conductivity for the two samples are very different.

**Figure 10:**
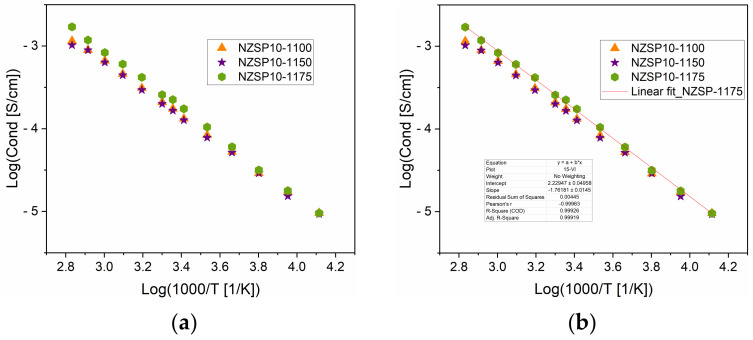
Variation in conductivity depending on temperature, for the samples NZSP10-1100, NZSP10-1150, and NZSP10-1175 (**a**); linear fit of conductivity values for NZSP10-1175 (**b**).

The values of ionic conductivity for the analyzed samples were determined from the graphs of the real conductivity for different temperatures. The ionic conductivities determined for samples with 10% Na, sintered at different temperatures are represented in Figure 10. The conductivity increases linearly with temperature (Figure 10a) in the range of −30 to 80 °C (243–353 K). The higher ionic conductivity values are obtained for the samples NZSP10-1175, above 10 °C (283 K). The slope of the linear fit of the conductivities (Figure 10b) could be used for the determination of the activation energy, using the Arrhenius equation (Equation (3)). Table 4 presents the activation energy calculated for NZSP, NZSP5, and NZSP10.

(3) σ=σ0exp(−EakBT)

where *σ* represent the conductivity in direct current (ionic conductivity), *E_a_* is the activation energy for ionic conduction process, *k_B_* is the Boltzmann constant, and *T* is the absolute temperature (K). Including the slope value (S) in the Boltzmann equation, the activation energy could be calculated from Equation (4), considering *k_B_* = 1.38 × 10^−23^ J and 1J = 1.602 × 10^−19^ eV:

(4) S=−EakB·103=−1.76

**Figure 11:**
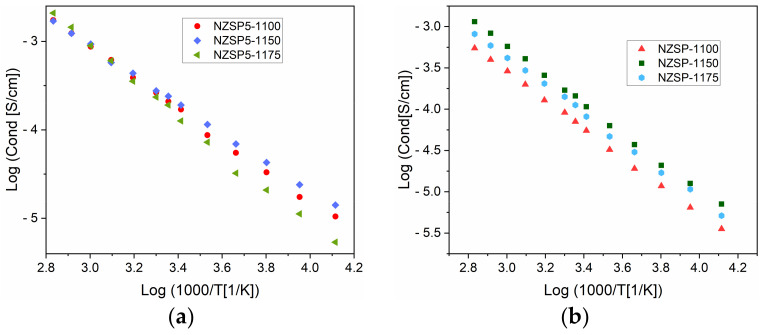
Variation in conductivity depending on temperature, for samples with NZSP5 (**a**) and NZSP (**b**), annealed at different temperatures.

In Figure 11, graphs of variation of the ionic conductivities are represented depending on temperature, for the samples NZSP and NZSP5 annealed at different temperatures. The linear trend can be noted for all samples, but the conductivity values at low temperatures are better for samples annealed at lower temperatures (NZSP5-1100 and NZSP5-1150. The conductivity of NZSP5-1175 exceeds the values of the other two samples, just above 60 °C.

**Figure 12:**
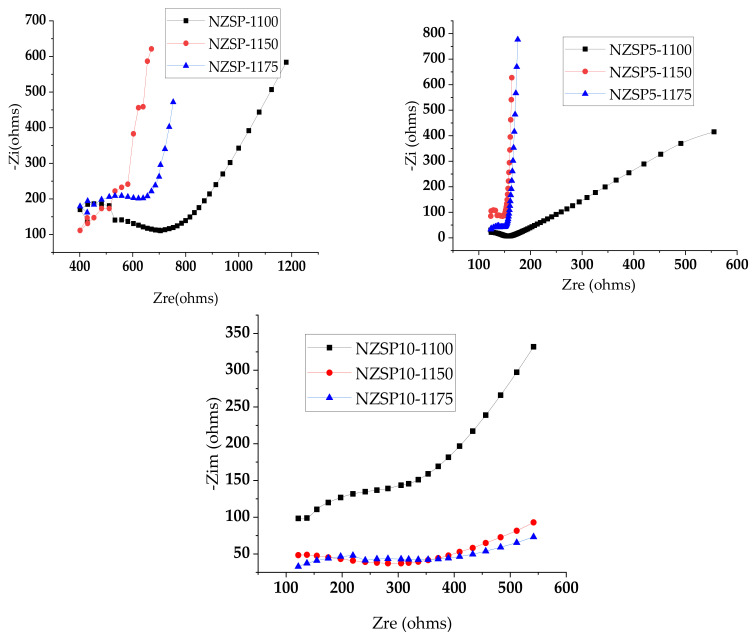
Nyquist plot NZSP, NZSP5, and NZSP10 samples at room temperature (RT) prepared at different sintering temperatures.

Figure 12 shows the Nyquist plot for the samples NZSP, NZSP5 and NZSP10, where the real and imaginary components of the measured conductivity are plotted in the broadband range from 10^−1^ to 10^6^ Hz, at room temperature. Conductivity measurements were performed by electrochemical impedance spectroscopy (EIS) in potentiostatic mode with Versa STAT 3F equipment (UK). The difference in amplitude and phase angle provides electrochemical information about the interfaces between the electrodes and the electrolyte. The current response is expressed as:

(5) $$I\left(t\right)=Iosin\;(\omega t-ϴ)$$

where *I*_0_ is the current amplitude and *θ* is the phase shift. Impedance is calculated by an expression analogous to Ohm’s law.

(6) $$Z=\frac{E(t)}{I(t)}=Zo\;(cos\omega +sin\omega )=Z\prime -jZ\prime$$

where *Z*′ is the real impedance, *Z*″ is the imaginary impedance, and *j* is the imaginary constant defined by *j*^2^ = −1

The EIS data were expressed by a Nyquist plot (Figure 12), which maps the real part of the impedance against the imaginary part of the impedance over a range of different frequencies. The Nyquist plots consisted of two semicircles in the high-frequency region and an oblique line in the low-frequency region, which are associated with the transport of Na^+^ in the electrolyte and the blocking effect of ions at the electrode–electrolyte interface, respectively.

## 4. Conclusions

In this work, NASICON ceramic membranes with the structure Na_3_Zr_2_Si_2_PO_12_, Na_3.15_Zr_2_Si_2_PO_12_ (5% Na excess), and Na_3.3_Zr_2_Si_2_PO_12_ (10% Na excess) were prepared using the classic system based on the solid-state reaction (SSR) synthesis method, by keeping the sintering temperature at the lower limit of 1100 °C, 1150 °C, and 1175 °C respectively. By dynamic light scattering (DLS) particle size measurement, 10% of the NASICON powder particles pre-sintered at 1100 °C were found to have a size of approx. 1000 nm, while for NASICON sintered at 1150 °C the particles have a size between 618.6 and 1315.4 nm, and for NASICON sintered at 1175 °C the particles have a size between 500.6 and 1008 nm. From the data obtained, it is observed that during sintering a change in the texture of the NASICON composition occurs as a direct consequence of the high sintering temperature. As the sintering temperature increases, so does the particle size. EDX analysis indicated a homogeneous distribution of elements which is observed from EDX mappings. The obtained curves indicated a hysteresis loop in the adsorption–desorption isotherms, suggesting the presence of a mainly mesoporous structure. Investigating the effects of isostatic pressure applied for the formation of NASICON granules, the pressure of 3000 kgF/cm^2^ produced the best results at sintering temperatures of 1150 °C and 1175 °C. Laboratory-prepared samples matched the ionic conductivity of the commercial reference 3.31 × 10^−4^ S/cm. Following the experiments for NASICON membranes without excess Na, with 5% excess Na, and with 10% excess Na, synthesized at different pressing forces and sintering temperatures, it was found that membranes with an excess 10% Na, sintered at 1175 °C for 10 h, presented the highest ionic conductivity (4.72 × 10^−4^ S/cm).

## Figures and Tables

**Table 1:** Particle size distribution for NASICON membranes.

Sample	D_10_ [nm]	D_50_ [nm]	D_90_ [nm]	D_m_ [nm]
NZSP Ref.	705.8	876.8	1109.9	861.2
**without Na excess**				
NZSP 1100	1033.8	1284.7	1626.8	1261.9
NZSP 1150	933.9	1681.0	2895.0	1400.8
NZSP 1175	500.6	881.9	1477.8	738.9
**Na excess 5%**				
NZSP-5 1100	910.5	1441.9	1457.4	1120.9
NZSP-5 1150	1315.4	1646.4	2097.9	1616.3
NZSP-5 1175	1008.5	1248.5	1575.8	1226.7
**Na excess 10%**				
NZSP-10 1100	1039.3	1290.7	1633.6	1267.9
NZSP-10 1150	618.6	779.3	980.1	765.9
NZSP-10 1175	762.9	949.6	1204.2	932.6

**Table 2:** Analysis of specific surface area (BET method), porosity (BJH method) for NZSP, NZSP5 and NZSP10.

Sample	Specific Surface ^1^ (m^2^ g^−1^)	Pore Volume ^2^ (cc g^−1^)	Mean Pore Radius ^2^ (Å)
NZSP	2.69	0.008	16.55
NZSP 5	2.94	0.043	18.52
NZSP 10	11.783	0.041	18.55

^1^ Analysis performed by the BET method; ^2^ Analysis performed by the BJH method.

**Table 3:** Activation energies (E_a_) of the ionic conductivity for the analyzed samples.

Samples	NZSP-1100	NZSP-1150	NZSP-1175	NZSP5-1100	NZSP5-1150	NZSP5-1175	NZSP10-1100	NZSP10-1150	NZSP10-1175
Slope	−1.73	−1.75	−1.72	−1.76	−1.64	−2.04	−1.65	−1.64	−1.76
E_a_ (eV)	0.149	0.1507	0.1481	0.1516	0.1412	0.175	0.1421	0.1412	0.1516

**Table 4:** Ionic conductivity and synthesis condition of Na_3_Zr_2_Si_2_PO_12_ at room temperature (RT), reported in the literature.

Sample	Sintering Condition (°C)	Pressing Force (kgF/cm^2^)	Ionic Conductivity (σ) at RT (S/cm)	Reference
Na_3_Zr_2_Si_2_PO_12_( NSZP)	1100 °C/10 h	3000	7.40 × 10^−5^	Present work
	1150 °C/10 h		1.47 × 10^−4^	
	1175 °C/10 h		1.55 × 10^−4^	
Na_3.15_Zr_2_Si_2_PO_12_ (NZSP 5)	1100 °C/10 h	3000	2.43 × 10^−4^	
	1150 °C/10 h		2.43 × 10^−4^	
	1175 °C/10 h		4.40 × 10^−4^	
Na_3.3_Zr_2_Si_2_PO_12_ (NZSP 10)	1100 °C/10 h	3000	1.20 × 10^−4^	
	1150 °C/10 h		1.25 × 10^−4^	
	1175 °C/10 h		4.72 × 10^−4^	
Na_3_Zr_2_Si_2_PO_12_-Ref (NAS Ref.)	-	-	3.31 × 10^−4^	
Na_3_Zr_2_Si_2_PO_12_	1125 °C/2 h	-	5.26 × 10^−4^	[1]
	1100 °C/6 h	-	3.18 × 10^−4^	
	1300 °C/4 h	-	2.02 × 10^−4^	
	1150 °C/12 h	-	4.56 × 10^−4^	
	1200 °C/12 h	-	3.35 × 10^−4^	
	1150 °C/5 h	-	1.10 × 10^−4^	
Na_3_Zr_2_Si_2_PO_12_ with 5% Na excess	1100 °C/12 h	-	1.10 × 10^−4^	[14]
Na_3_Zr_2_Si_2_PO_12_	1220 °C/10 h	-	1.10 × 10^−4^	
Na_3_Zr_2_Si_2_PO_12_ (excess Na and P)	1100 °C/12 h	-	3.72 × 10^−4^	[27]
	1150 °C/12 h	-	3.86 × 10^−4^	
	1200 °C/12 h	-	4.57 × 10^−4^	
	1280 °C/12 h	-	9.53 × 10^−5^	
Na_3_Zr_2_Si_2_PO_12_ (excess Na)	1100 °C/12 h	-	1.13 × 10^−3^	
	1150 °C/12 h	-	5.83 × 10^−4^	
	1200 °C/12 h	-	3.70 × 10^−4^	
	1280 °C/12 h	-	2.46 × 10^−5^	
Na_3_Zr_2_Si_2_PO_12_	1280 °C/10 h	-	1.03 × 10^−3^	
	1000 °C	-	2.2 × 10^−4^	
	1050 °C	-	3.3 × 10^−4^	
	1100 °C	-	3.6 × 10^−4^	
	1150 °C	-	4.6 × 10^−4^	
	1200 °C	-	2.4 × 10^−4^	
Na_3_Si_2_Zr_1.88_Y_0.12_PO_12_	1190 °C/10 h	-	4.6 × 10^−3^	[28]
	1210 °C/10 h	-	4.6 × 10^−3^	
	1220 °C/2 h	-	4.1 × 10^−3^	
	1220 °C/10 h	-	4.7 × 10^−3^	
	1220 °C/40 h	-	6.6 × 10^−3^	
	1220 °C/80 h	-	1.6 × 10^−3^	
	1230 °C/10 h	-	4.5 × 10^−3^	
	1235 °C/10 h	-	2.2 × 10^−3^	
Na_3_Zr_2_ Si(O_4_)_2_PO_4_	1250 °C/5 h	1500	1.1 × 10^−3^	[29]
Na_3.4_Zr_2_Si_2.4_P_0.6_O_12_	-	-	5.2 × 10^−3^	[30]
Na_3.2_Zr_2_Si_2.2_P_0.8_O_12_	-	-	3.6 × 10^−3^	
Na_3.1_Zr_1.55_Si_2.3_P_0.7_O_11_	1250 °C/1 h	-	9.0 × 10^−4^	[31]
Na_3_Zr_2_Si_2_PO_12_	1250 °C/4 h	2039	2.02 × 10^−4^	[32]

## Data Availability

Data are contained within the article.
